# Preparation and Performance of Repair Materials for Surface Defects in Pavement Concrete

**DOI:** 10.3390/ma16062439

**Published:** 2023-03-18

**Authors:** Pengfei Li, Zhongyang Mao, Xiaojun Huang, Min Deng

**Affiliations:** 1 College of Materials Science and Engineering, Nanjing Tech University, Nanjing 211800, China; 202061203171@njtech.edu.cn (P.L.);; 2 State Key Laboratory of Materials-Oriented Chemical Engineering, Nanjing 211800, China

**Keywords:** surface defect, repair material, ethyl acetate, infiltration performance

## Abstract

Concrete surface defects are very complex and diverse, which is a great test for repair materials. The efficiency and durability of the repair system depend on the bonding effect between the concrete and the repair material. However, the rapid increase in system viscosity during the reaction of repair materials is an important factor affecting the infiltration effect. In the present work, the infiltration consolidation repair material was prepared, and its basic properties (viscosity, surface drying time and actual drying time, infiltration property) and mechanical properties were evaluated. Finally, the infiltration depth, film-forming thickness, and anti-spalling ability of concrete under a single-side freeze–thaw cycle are revealed. The results showed that using ethyl acetate could rapidly reduce the viscosity of the repair material, and the repair material could penetrate 20–30 mm into the concrete within 10 min. It was found by laser confocal microscopy that the thickness of the film formation after 3 days was only 29 µm. In the mortar fracture repair test to evaluate the bond strength, the bond strength of the repaired material reached 9.18 MPa in 28 days, and the new fracture surface was in the mortar itself. In addition, the freeze–thaw cycle test was carried out on the composite specimens under salt solution to verify the compatibility of the designed repair material with the concrete substrate. The data showed that the average amount of spalling was only 1704.4 g/m^2^ when 10% ethyl acetate was added. The penetrating repair material in this study has good infiltration performance, which can penetrate a certain depth in the surface pores and form a high-performance consolidation body, forming a “rooted type” filling.

## 1. Introduction

With the dramatic increase in traffic volume in recent years, concrete pavement bears more and more load. The long-term load and impact pressure, coupled with the destruction of the natural environment (freeze–thaw cycles, dry–wet cycles, corrosive substances), concrete pavement withstands varying degrees of damage [1]. Once the defects appear, they provide a channel for the corrosive medium, aggravate the failure of the concrete, and seriously reduce the service quality of the pavement [2,3,4]. Excavating and re-paving the damaged road surface inevitably causes a waste of manpower and material resources as well as an inconvenience to open traffic and fast navigation [5,6]. Therefore, it is an urgent problem to repair concrete pavement quickly and efficiently. Selecting appropriate repair materials can effectively delay the sustainable development of concrete micro-cracks and improve the durability of concrete, which requires the repair materials to have low viscosity, excellent mechanical properties, and strength stability [7,8,9].

At present, the materials used for concrete repair are mainly divided into three categories. The first category is inorganic repair materials which mainly include silica cement, fast-hardening Portland cement, magnesium phosphate cement, and so on [10,11]. The second category is organic repair materials which mainly include epoxy resin, acrylic resin, polyurethane, and acrylamide [12]. The third category is polymer repair materials compounded by organic and inorganic materials, for example, vinyl polyester acid emulsion modified cement mortar, propyl cement mortar, and epoxy mortar concrete [13,14]. However, there are many kinds of repair materials, and they all have different advantages and disadvantages.

The research on concrete repair materials now focuses on good compatibility with concrete, good durability, and low viscosity for easy grouting and repair. Kim et al. [15] used a combination of epoxy resin injection and surface pretreatment by a dipping agent. For the freeze–thaw cycle test, both the relative dynamic elastic modulus and durability index were greater than 80% after 300 cycles when the exposure ended for all the specimens, and the chloride resistance test results confirmed that repair methods, including impregnation, had a penetration effect 2–3.5 times greater than the epoxy repair method. Hassan et al. [16] used an acrylic copolymer and a cement/aggregate/admixture mixture to form a polymer-modified repair mortar (PMC). The PMC repair mortar showed the most appropriate properties in terms of dimensional stability with concrete due to a similar elastic modulus and low shrinkage strains when compared to the parent concrete. Khalina et al. [17] studied the effect of aliphatic reactive diluents on the properties of two different epoxy resins, bifunctional and multifunctional epoxy resins. It was found that as the diluent was gradually incorporated into the epoxy resin, the damage mode of the epoxy resin specimens changed from brittle to more ductile under the bending test. The fracture strain of the bifunctional resin increased by 75%, and that of the multifunctional resin increased by more than 24%. The diluent content enhanced the fracture toughness of the epoxy resin by about 20% and 29% for the bifunctional and multifunctional resins, respectively.

As surface defects contain many tiny cracks, it is difficult for high-viscosity organic repair materials to penetrate the surface layer to a certain depth. Moreover, the higher the viscosity, the poorer the wettability of repair materials, most of which remain on the concrete surface to form thick polymers [18,19,20,21]. At present, the viscosity of organic repair materials is high, and it is difficult to penetrate a certain depth of concrete surface, and it is unable to complete the effective plugging of pores [22]. In addition, the surface cures to form a thick polymer film, which is easily removed, resulting in a poor repair effect. Therefore, reducing the viscosity of the prepolymer is important to ensure excellent permeability.

In view of this, tetrachloroethylene was selected as a shrinkage reducer [23], and anhydrous ethanol, ethyl acetate, and acetone were used as inactive diluents. The influence of diluent addition on the material properties (viscosity, grouting depth, gel time) was clarified, and a series of studies were completed on the mechanical properties of the materials. This paves the way for penetrating repair materials to repair pavement defects.

## 2. Materials and Methods

### 2.1. Raw Materials

The design strength grade of an ordinary concrete specimen is C30, in which the cement is Conch brand P·O 42.5 ordinary Portland cement. The aggregate is a well-graded crushed stone with a particle size of less than 30 mm and a fineness modulus of 2.5 medium sand. See Table 1 for the specific mix ratio.

Methyl methacrylate (MMA) is the main raw material, dibenzoyl peroxide (BPO) is the initiator, dibutyl phthalate (DBP) is the plasticizer, tetrachloroethylene (PCE) is the shrinkage agent, N,N-dimethylaniline (DMA) is the curing agent, and the three diluents are absolute ethanol, ethyl acetate, and acetone. Table 2 shows the proportion of raw materials used to prepare MMA-based repair materials.

### 2.2. Preparation of the Penetrating Repair Materials

1. Preparation of the MMA-based repair materials

MMA is used as the main raw material, BPO as the catalyst, DBP as the plasticizer, and PCE as the shrinkage agent to modify the repair material. The preparation process is as follows.

- A certain amount of MMA was poured into a beaker, stirred at a speed of 300/min, and distilled in a water bath at 50 °C for 10 min.
- After distillation to remove the inhibitor, 1.5% BPO, 30% DBP, and 10% PCE was added and continuously stirred for 2 min.
- The curing agent DMA was added to the prepolymer and stirred evenly until the set time of the test was reached. The three-flask was quickly put into cold water to slow down the polymerization reaction, and the MMA-based repair material was obtained.

2. Preparation of the MMA-based penetrating repair materials

Infiltration-type repair materials penetrate a certain depth of concrete surface by self-weight. In order to ensure good infiltration performance of the repair materials, thinner is used to reduce the viscosity of the repair materials, and DMA is used to promote curing to prepare the penetrating repair materials. The preparation process is as follows.

- The configured prepolymer was added with a certain amount of DMA and stirred for 2 min at 300 r/min.
- After an average of 60 min, diluent was added and stirred at 300 r/min for 3 min to obtain permeable repair material.

### 2.3. Pretreatment of the Concrete Specimen

**Figure 1:**
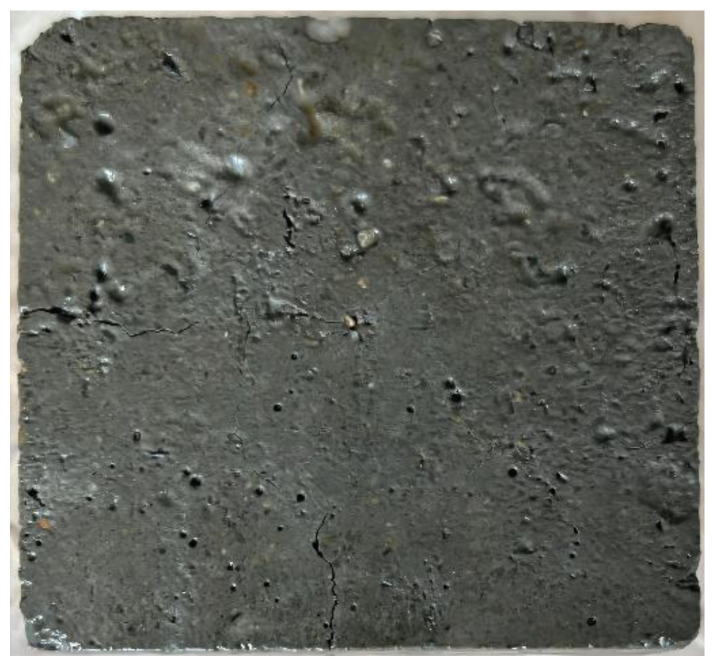
Surface of concrete specimen after pretreatment.

A number of 100 × 100 × 100 mm concrete specimens were prepared and placed in a laboratory with a temperature of (20 ± 2) °C and a relative humidity of (65 ± 5)% for 28 days. The cured specimens adopt the single-side freeze–thaw method according to the long-term performance and durability test method of ordinary concrete (GBT50082-2009) so that the infiltration surface of the repaired material is closer to the damaged concrete with surface defects. The specific specimen is shown in Figure 1.

### 2.4. Test Methods

#### 2.4.1. Viscosity Test

**Figure 2:**
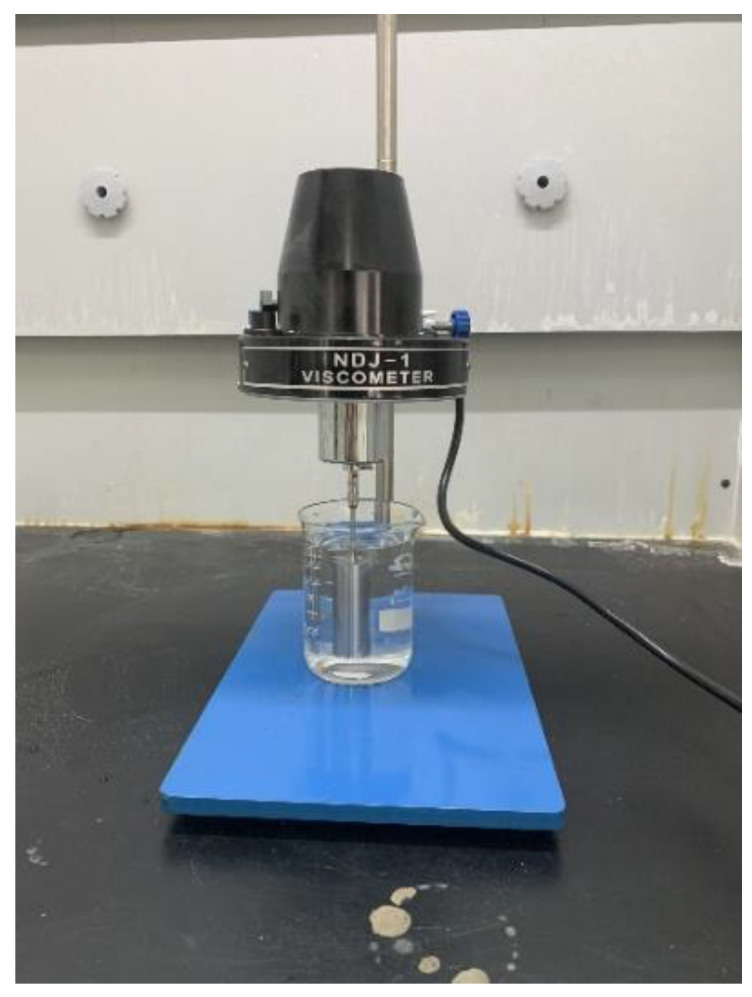
Pointer rotary viscometer NDJ-1.

The initial viscosity of the repair material has a great influence on the infiltration performance. The smaller the viscosity of the repair material, the stronger its fluidity. In this study, the NDJ-1 rotary viscometer (Shenzhen, China) was used to measure the viscosity of the repair material. The viscometer equipment is shown in Figure 2. By analyzing the ratio of raw materials in Table 2, the initial viscosity of the repair material was measured at 20 °C ± 2 °C, and the average viscosity of 60 min was used as the evaluation index.

#### 2.4.2. Surface-Drying Time and Through-Drying Time

The surface-drying time is determined according to the provisions of GB/T 1728-2020 method B (refers to trigger), and the surface of the coating film is considered dry when no paint adheres to the finger after touching the surface of the coating film. The through-drying time is determined according to the provisions of GB/T 1728-2020 in method C (blade method), and the coating film is considered dry by cutting and scraping the coating film on the template with a blade and observing that there is no adhesion phenomenon in the bottom layer and film.

#### 2.4.3. Determination of the Film-Forming Thickness

**Figure 3:**
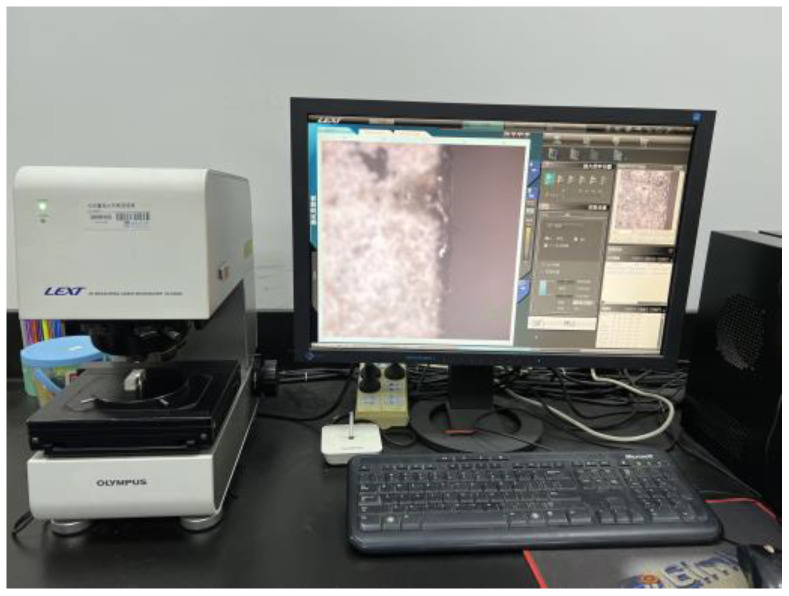
Laser confocal microscope.

Film forming thickness is an important index to evaluate the surface properties of the tunnel. The thinner the film thickness is, the less influence it has on the original excellent friction property of the concrete surface. In this study, a laser confocal microscope (OLYMPUS, Japan) was used to observe a section of the concrete specimen after 6 h, 24 h, and 3 d of curing of the coated repair material at 25 °C. The equipment is shown in Figure 3. The curing specimens were cut in the middle, and the average thickness of five uniform points in the section was taken as the film thickness.

#### 2.4.4. Infiltration Performance

The infiltration performance of the repair material is evaluated by measuring the infiltration depth. A 100 × 100 × 100 mm concrete specimen is used, and the concrete surface is first cleaned of floating ash before applying the repair material. The repair material is applied three times with a 10-min interval between each application. The repaired specimen is then cured at room temperature for 3 d. The concrete block is cut, and the infiltration of the solidified repair material is observed from the cut surface. Three points with relatively uniform infiltration are selected, and the infiltration depth at each point is observed. The average of the infiltration depths at the three points is taken as the final infiltration depth of the repair material on the surface of the concrete.

#### 2.4.5. Bond Strength

**Figure 4:**
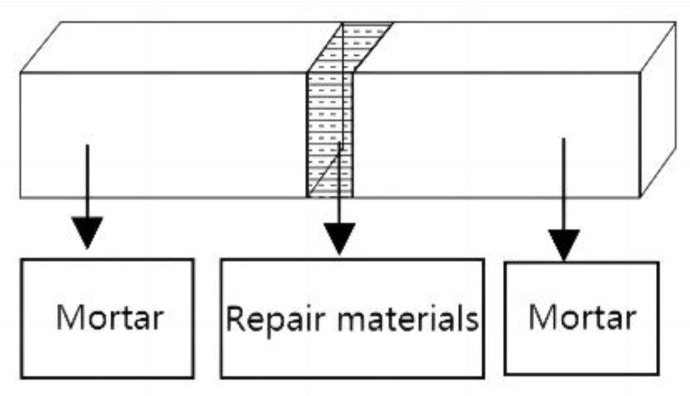
The model view of specimen for bond test.

Using P·O 42.5 cement, mortar specimens with dimensions of 40 × 40 × 160 mm were prepared. The specimens were cured under standard conditions (temperature of (20 ± 2) °C and relative humidity of 95%) for 28 d. After curing, the specimens were placed in a drying oven at 105 °C for 6 h to serve as the dry interface specimens for bond strength testing. The specimens that had completed standard curing were used as the wet interface specimens for bond strength testing. The mortar flexural strength testing machine was used to break the specimens, and according to the Japanese standard JISA6024-1998, a gap of 3~5 mm was left between the two broken sections of the specimen and fixed in place. MMA prepolymer was then injected into the gap [24]. As shown in Figure 4, after being cured in a 60 °C oven for 4 h, the repaired material experienced some volume shrinkage. Therefore, the cracks were filled again with MMA prepolymer. The flexural strength of the specimens was tested, and the fracture location was observed after being cured at room temperature (temperature of (20 ± 2) °C and relative humidity of 60%) for 3 d, 7 d, and 28 d.

#### 2.4.6. Freeze–Thaw Cycle Test

**Figure 5:**
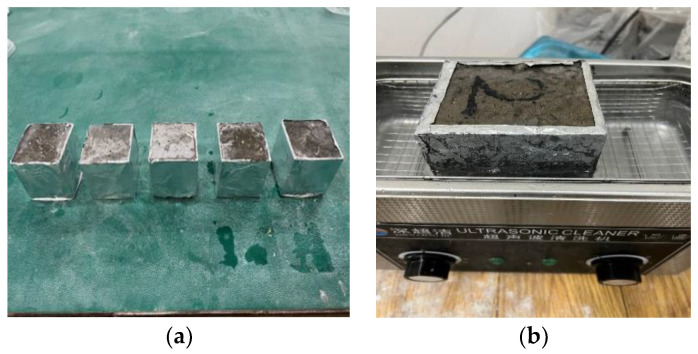
Preparation of freeze–thaw specimens: (**a**) prepared specimen; (**b**) ultrasonic cleaning.

The freeze–thaw cycle test after concrete repair can reflect the performance of the repaired material in an environment of large temperature difference [25]. According to the long-term performance and durability of ordinary concrete test method GBT50082-2009, this test adopts a single-side freeze–thaw method. The specimens that have reached the specified curing age are placed in a laboratory with a temperature of (20 ± 2) °C and a relative humidity of (65 ± 5)% to dry for 28 d. The side of the 100 × 100 × 100 mm specimen is coated with Sikaflex neutral silicone sealant and sealed with aluminum foil paper. All sides are sealed to prevent side peeling and ensure that the specimens are in a single-sided water absorption state. When adding the test liquid (3% NaCl solution) to the test container, care was taken to avoid wetting the top of the specimen, which is in a capillary absorption state. The specimens are immersed in NaCl solution to a depth of about 10 mm. Each group of tests consists of 5 specimens, and measurements are taken every four cycles. Ultrasonic cleaning is used to collect the peeling materials, as shown in Figure 5a,b for specific operating steps.

The calculation and treatment of test results comply with the following provisions:

(1) $$\mu_{s}=\mu_{b}-\mu_{f}$$

The mass of crushed concrete debris on the surface of the specimen $\mu_{s}$ is calculated according to equation (1), where $\mu_{s}$ is the quality of concrete slag on the specimen surface; $\mu_{f}$ is the quality of filter paper; and $\mu_{b}$ is the total mass of filter paper and concrete slag after drying.

After N freeze–thaw cycles, the total mass of detached particles per unit testing surface area of an individual specimen is calculated using Equation (2).

(2) $$m_{n}=\;\frac{\sum \mu_{s}}{A}\times 10^{6}$$

where $m_{n}$ is the N freeze–thaw cycles, single specimen unit test surface area total mass of exfoliation (g/m^2^); $\mu_{s}$ is the spalling mass of the specimen obtained from each test gap; and A is the surface area of a single specimen test surface (${\mathrm{mm}}^{2}$).

The arithmetic mean value of the total spalling mass per unit area of each group of specimens was taken as the measurement value of the total spalling mass $m_{n}$.

#### 2.4.7. SEM

**Figure 6:**
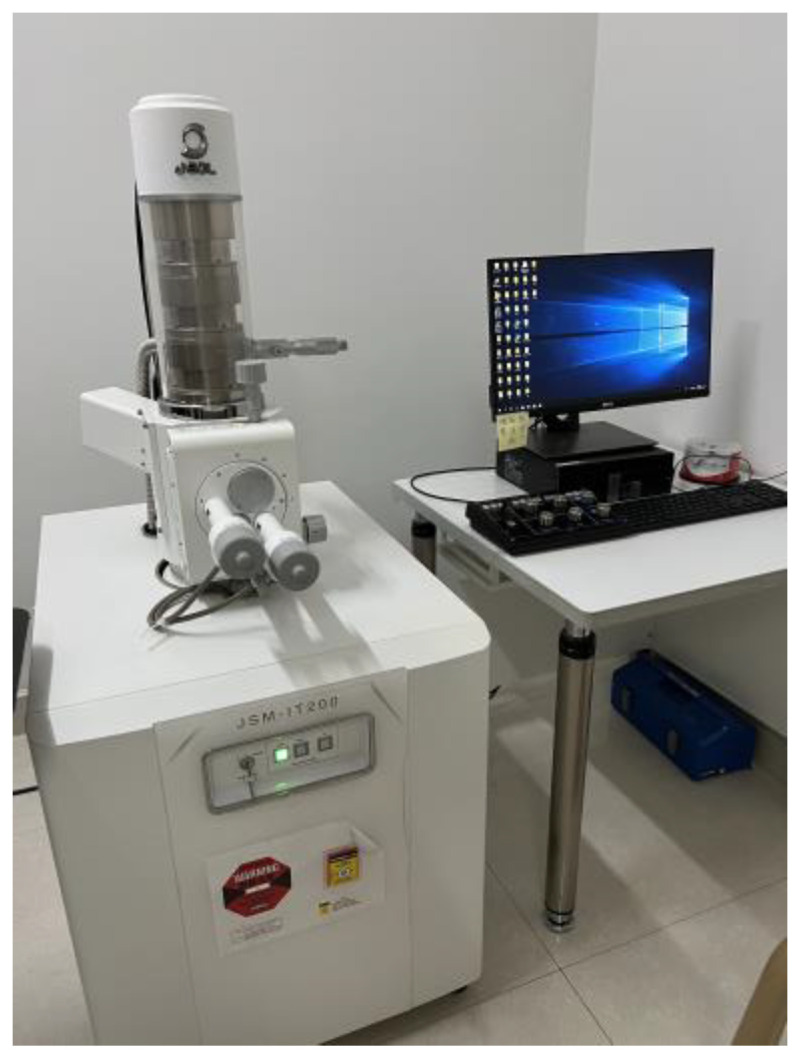
Scanning electron microscope.

A JSM-IT200 scanning electron microscope (Beijing, China) was used to observe the high-resolution microstructure of cement mortar. The equipment is shown in Figure 6. The samples used were taken from the broken parts of the mortar surface and scanned point by point with a focused high-energy electron beam, magnifying 10–100,000 times.

## 3. Results and Discussion

### 3.1. Preparation Technology of the MMA-Based Repair Materials

In this study, a curing agent was mainly added to accelerate the curing of repair materials. Therefore, the content of the curing agent has a great impact on the properties of the repair materials. In order to obtain the best preparation technology for infiltration consolidation repair materials, the average viscosity at 60 min, surface drying time, and tensile strength were used as evaluation indexes. The experiment investigated the addition of 0.3%, 0.5%, 0.7%, and 1% of the curing agent to the prepolymer with the proportion of MMA 60, BPO 1.5%, DBP 30%, and PCE 10%.

#### 3.1.1. Effect of Preparation Technology on the 60-Min Average Viscosity

**Figure 7:**
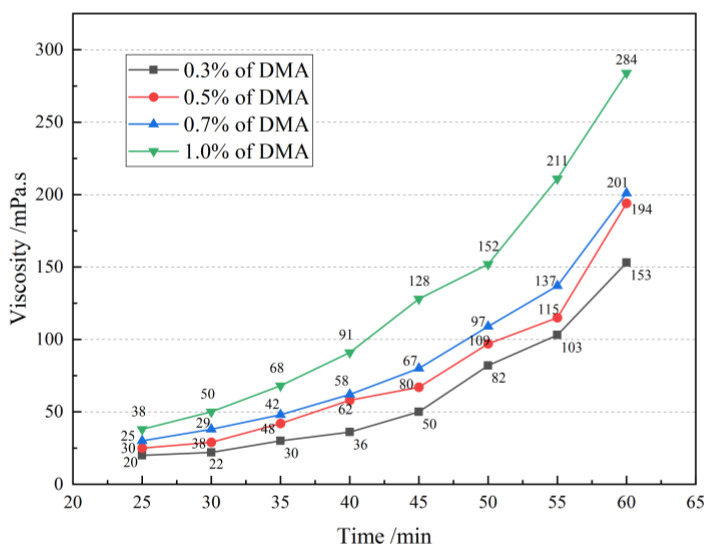
60-min average viscosity range.

The initial viscosity of the repair material is an important factor affecting the repair effect. Using the average viscosity after 60 min of adding the curing agent as the evaluation index, Figure 7 shows the variation range of the average viscosity of the repair material after adding different amounts of curing agent.

As shown in Figure 7, the 60-min average viscosity of the repair material generally showed a slow-then-fast trend. In the early stage of polymerization, when the synthesis time is short, the number of activated centers for MMA monomers is small, and the polymerization chains are not long, resulting in a small polymerization rate. Therefore, the viscosity of the repair material does not change much with increasing synthesis time. At a reaction time of 25 min, the viscosity of the repair material with 0.3% DMA content is 20 mPa·s, while that of the repair material with 1% DMA content is 38 mPa·s. Within the time interval of 25 to 40 min, the viscosity curve of the material shows a steady increase, maintaining below 100 mPa·s. As the reaction progresses from 40 to 60 min, the viscosity of the system increases significantly. The viscosity of the repair material with 1% DMA content increases sharply, and the average viscosity at 60 min reaches 284 mPa·s, which is a 46.12% increase compared to the average viscosity of 153 mPa·s for the repair material with 0.3% DMA content. Therefore, DMA content has a significant impact on the viscosity of the repair material. When the concentration of the curing agent is too high, the polymerization reaction rate will accelerate, generating a large amount of heat. If it cannot dissipate in time, the viscosity will suddenly increase, causing explosive polymerization, seriously affecting the repair effect.

#### 3.1.2. Effect of Preparation Technology on the 60-Min Drying Time

**Figure 8:**
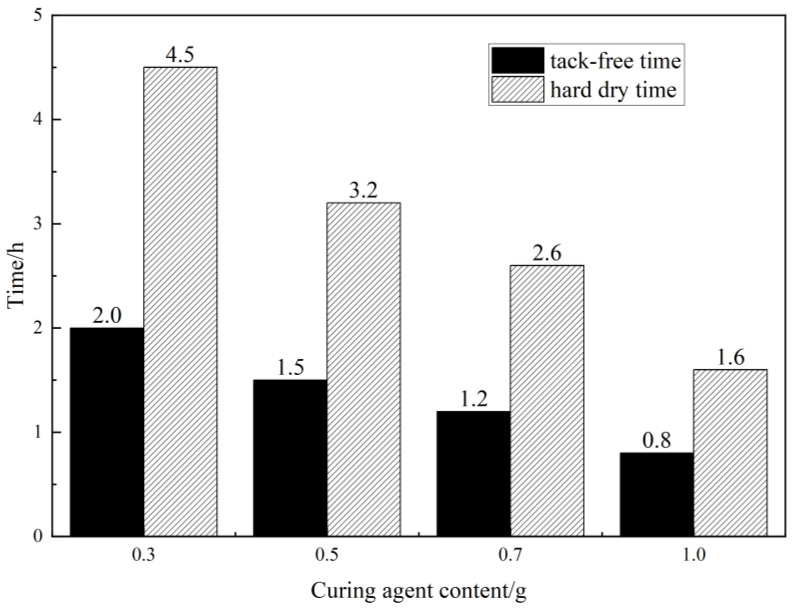
Influence of curing agent content on drying time.

The drying time of the repair material directly affects the work performance and can also greatly shorten the construction period. The prepolymer is applied to the template and cured at 25 °C. The drying time and actual drying time of the repair material were discussed, and the effect of different curing agent dosages on the drying time and actual drying time of the material was analyzed, as shown in Figure 8.

From Figure 8, it can be observed that the drying time and actual drying time of the material decrease with an increase in the curing agent content. When the DMA content is 1%, the drying time and actual drying time of the repair material reach 0.8 h and 1.5 h, respectively, which are 60 and 67% shorter than the drying time and actual drying time of the repair material with 0.3% DMA content. It can be seen that the proportion of the curing agent in the MMA-based repair materials is the main factor affecting the curing rate of the system. When the amount of curing agent added is too small, it cannot participate in the reaction fully, or the performance of the cured product is poor and cannot meet the requirements. On the other hand, when the amount of curing agent added is too large, the curing rate of the system increases significantly, resulting in the production of a large number of bubbles during curing, which seriously affects the physical properties of the material itself. Therefore, in practical applications, the amount of curing agent should be adjusted reasonably according to different usage conditions, which can achieve a dual adjustment of the curing rate and the performance of the cured product [26].

#### 3.1.3. Effect of Preparation Technology on the 60-Min Bond Strength

**Figure 9:**
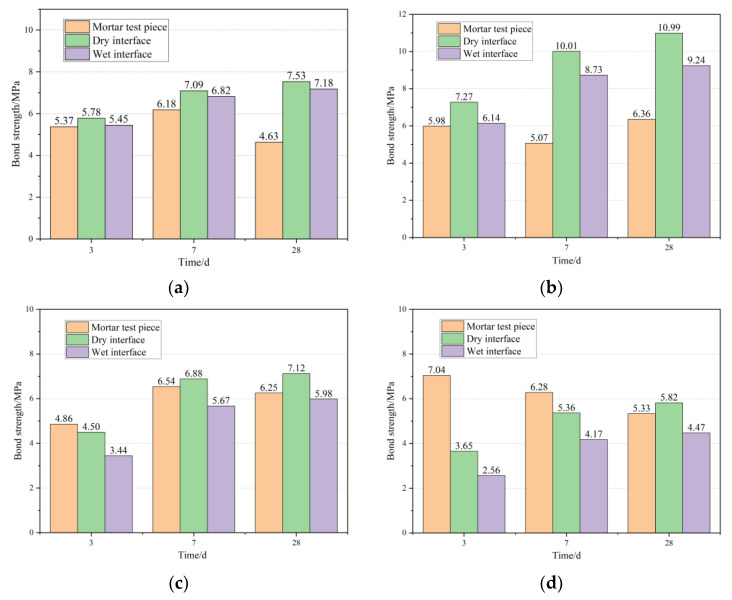
Relationship between wet and dry interface and bond strength: (**a**) 0.3% DMA; (**b**) 0.5% DMA; (**c**) 0.7% DMA; (**d**) 1% DMA.

After filling the cracks with prepolymers containing 0.3%, 0.5%, 0.7%, and 1% mass fraction of the curing agent using the bonding strength test method described in 2.4.7, the repaired specimens were cured for a certain period of time and then subjected to a flexural strength test. The location of the fracture surface was observed, and the average flexural strength of three specimens for each dosage was taken as the final result of the bonding strength of the repair material, which was compared with the strength of mortar when broken. The test results are shown in Figure 9a–d, corresponding to different dosages of the curing agent.

It can be seen from Figure 9 that the bonding strength of the repair material at the dry interface and wet interface mostly exceeds the flexural strength of the mortar specimens themselves when broken. When the content of the curing agent is 0.3%, the bonding strength of the repair material at the dry interface and wet interface after 28 days is 7.53 Mpa and 7.18 Mpa, respectively. When the content of the curing agent is 0.5%, the peak bonding strength of the repair material at the dry interface and wet interface after 28 days is 10.99 Mpa and 9.24 Mpa, respectively. As the content of the curing agent increases, the bonding strength of the repair material gradually decreases. When the curing agent content is 1%, the bonding strength of the repair material on the dry interface and the wet interface after 28 days decreases to 5.82 Mpa and 4.47 Mpa, respectively, which is reduced by 47.0% and 51.6% compared with the 0.5% content. This indicates that with the increase in the curing agent content, the bonding strength of the repair material first reaches a peak and then gradually decreases.

The comparison of the bond strength of the repair material on the dry and wet interfaces shows that the bond strength on the wet interface is reduced by 3.8 to 29.9% compared to that on the dry interface. This indicates that the surface moisture of concrete directly affects the repair effect of the MMA-based repair materials. The presence of surface moisture affects the degree of curing of the material at the interface and reduces the roughness of the concrete interface, resulting in a decrease in the adhesion of the repair material to the concrete and a decrease in the bond strength. In contrast, on the dry interface, the penetrating repair material can penetrate the concrete to a certain depth, and the acrylic resin molecules can wrap and anchor the concrete, thereby enhancing the mechanical strength of the concrete to a certain extent [27].

### 3.2. Optimization of the Composition for the Penetrating Repair Material

Based on the optimal preparation process of MMA-based repair materials determined in Section 3.1, the average viscosity, drying time, and bonding properties of the repair materials with a DMA content of 0.3%, 0.5%, 0.7%, and 1% were comprehensively tested to further elucidate the variation law of material properties with DMA content. To ensure good infiltration performance of the repair material, a diluent was added to reduce the initial viscosity of the material, allowing it to penetrate the concrete surface to a certain depth without participating in the curing reaction.

#### 3.2.1. Penetration Depth

**Figure 10:**
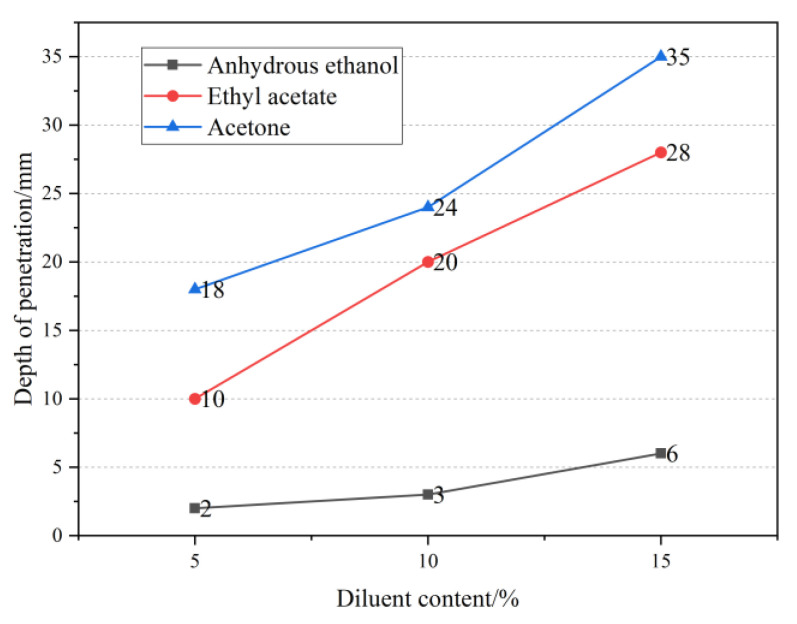
The influence of different diluent contents on the penetration depth.

To determine the trend of permeability of the MMA-based repair materials under different diluents, the effect of diluent type and content on the permeability of the repair materials was analyzed based on the determined optimal preparation process. The results are shown in Figure 10.

**Figure 11:**
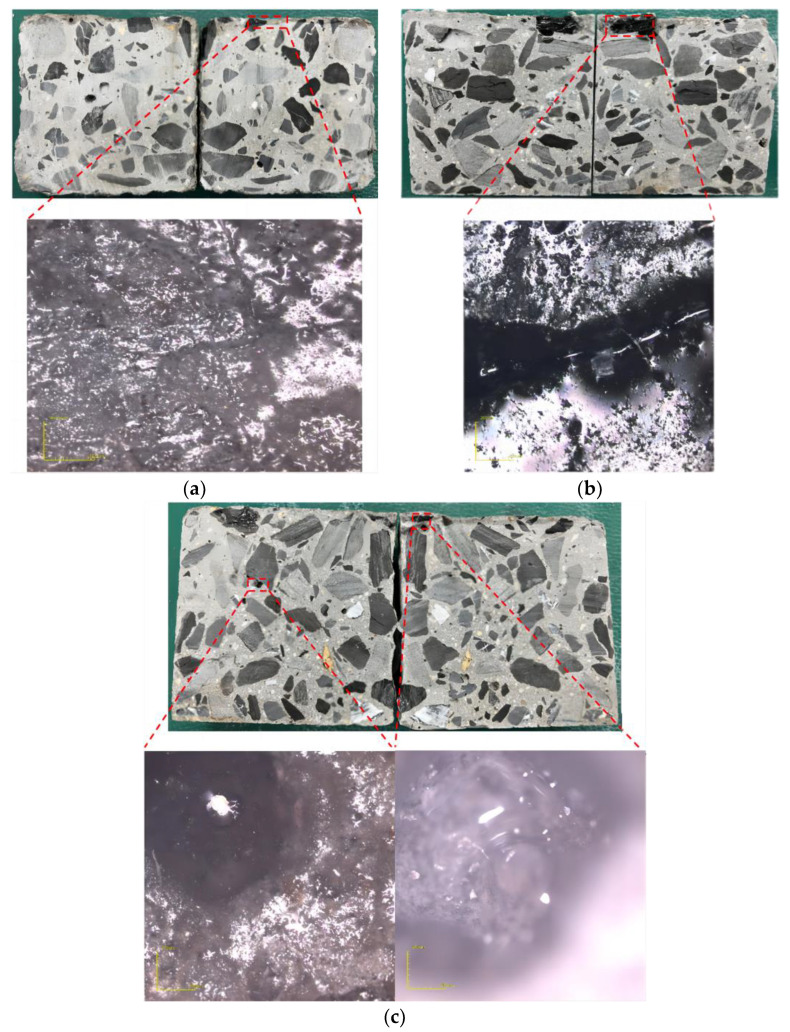
Infiltration condition: (**a**) anhydrous ethanol; (**b**) ethyl acetate; (**c**) acetone.

As shown in Figure 10, the penetration depth of the three diluents increased with the increase in their content. The penetration depth of the three diluents at 5% content ranged from 2–18 mm, while at 10% content, it ranged from 3–24 mm. When the content increased to 15%, the penetration depth reached 6–35 mm. This indicates that increasing the diluent content can increase the penetration depth of the repair material. The presence of the diluent reduces the concentration of the system, and the viscosity of acetone at room temperature is 0.31 mPa·s, which significantly reduces the viscosity of the repair material system, enabling it to penetrate the concrete surface layer up to 35 mm under specified conditions. The viscosity of anhydrous ethanol at room temperature is 1.17 mPa·s. Due to the hydrophobic interaction between PMMA and anhydrous ethanol, when they are mixed, non-covalent binding occurs between PMMA particles, resulting in a white flocculent matter that severely affects the penetration performance of the repair material. In the mixture, PMMA is absorbed and forms white crystalline PMMA. Generally, at the same temperature, the initial viscosity has a critical impact on the infiltration performance of the material. Therefore, repair materials containing acetone and ethyl acetate can infiltrate 10–35 mm deep into the concrete, effectively sealing the pore structure on the concrete surface and improving its mechanical properties. Figure 11a–c shows the penetration of the repair materials with different diluents in concrete and the cross-sectional laser microscope images. It can be seen that the repair materials exist in the microcracks and pores of the concrete, penetrate to a certain depth on the surface, and form a high-performance solid structure, forming a “rooted type” filling.

#### 3.2.2. Surface Film Thickness

**Figure 12:**
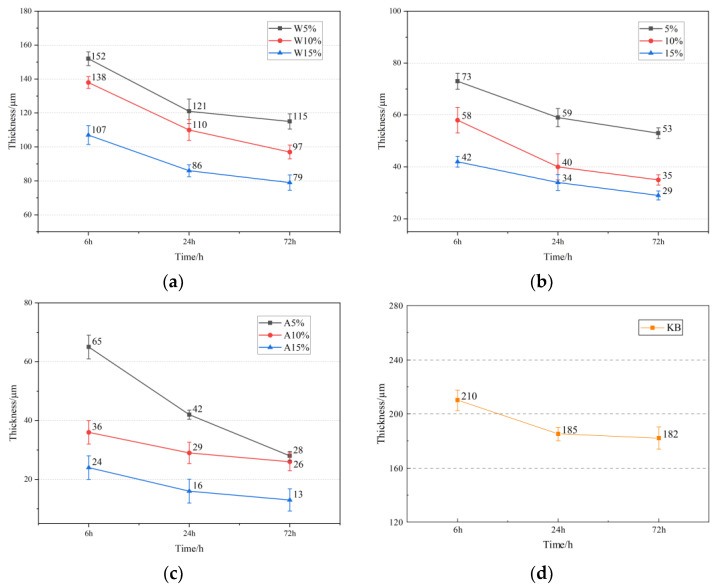
Effect of different diluents on film thickness: (**a**) anhydrous ethanol; (**b**) ethyl acetate; (**c**) acetone; (**d**) control.

**Figure 13:**
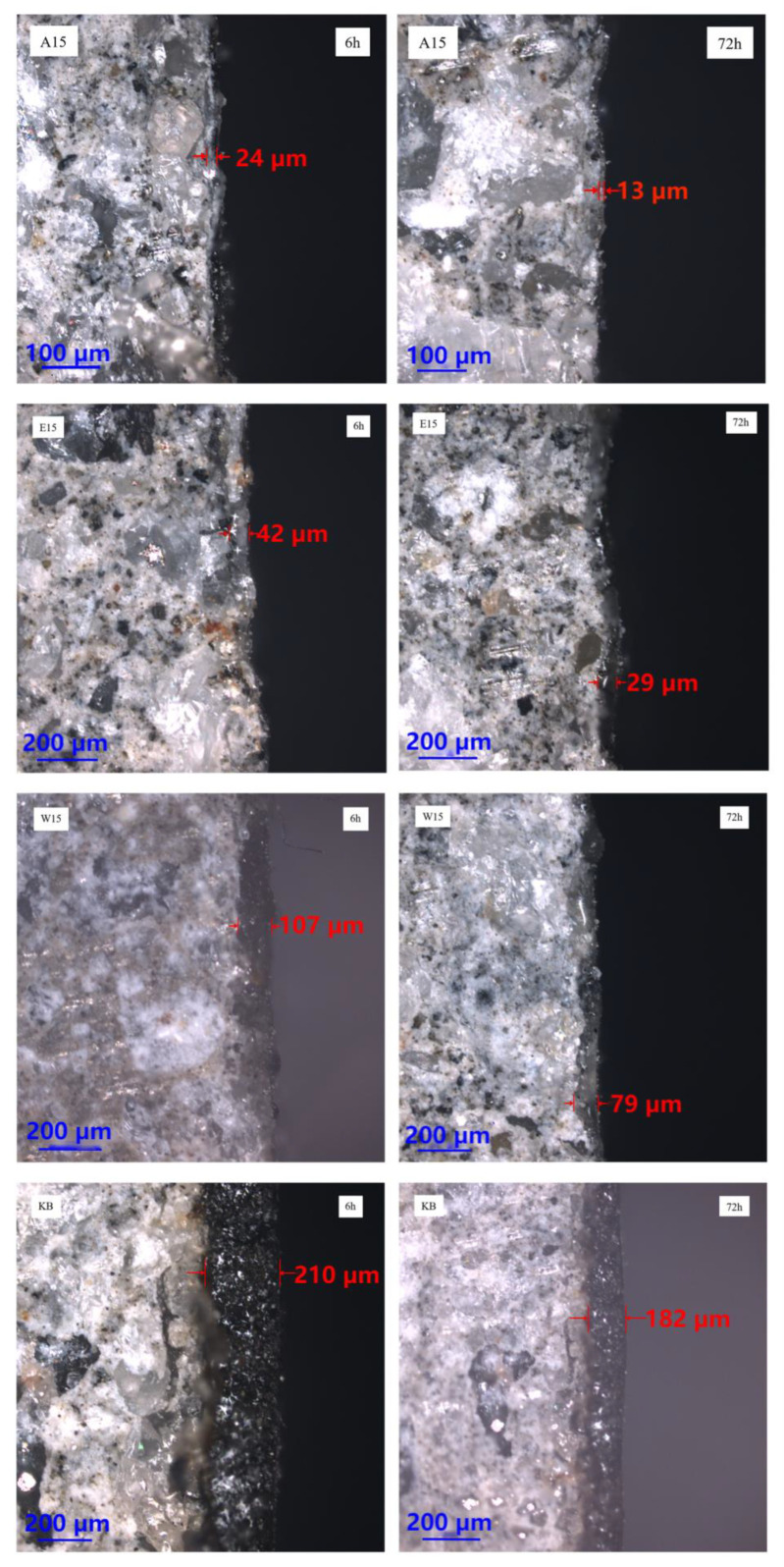
Results observed under a laser confocal microscope.

In order to elucidate the effect of the three diluents on the film thickness of the repaired concrete surface, blank samples and infiltrated solidified repair materials with different proportions were prepared. The film thickness of the repair material on the concrete surface was measured using laser confocal microscopy at 6 h, 24 h, and 72 h after preparation. The samples that were not completely cured and those that were fully cured were compared. The results are shown in Figure 12a–d and Figure 13.

From Figure 12a–d, it can be seen that with the increase in time, the thickness of the repair material added with three different diluents showed a trend of rapid decrease followed by a slow decrease during the curing process. The thickness of W10, E10, and A10 after 72 h were 97 μm, 35 μm, and 26 μm, respectively, which decreased by 46.7%, 80.8%, and 85.7% compared to the repair material without diluent. The results showed that the addition of acetone resulted in the most significant reduction in film thickness, and the repair materials with diluents were able to penetrate the surface of the concrete well, reducing the film thickness on the surface and significantly reducing the impact on the excellent friction performance of the concrete surface. The fundamental reason for this lies in the low initial viscosity and high surface tension of the repair material in the first stage. Capillary action occurs between the material and the concrete capillary pores, and the additional pressure generated by the capillary action provides the driving force for penetration during the penetration process [28]. The greater the surface tension of the material and the smaller the contact angle between the material and the concrete, the more favorable it is for the material to penetrate the concrete structure [29]. In the second stage, as the oxidation–reduction reaction of the material continues, the already penetrated material gradually solidifies and forms a “rooted type” filler, while the material that has not penetrated accumulates and solidifies on the concrete surface to form a polymer film.

Figure 13 shows the comparison photos of thickness taken by laser confocal microscope. It can be clearly seen that the thickness of W15, E15, A15, and KB after 72 h are 79 μm, 29 μm, 13 μm, and 182 μm, respectively, which are decreased by 26.2%, 30.9%, 45.8%, and 13.3% compared to the thickness after 6 h. The results show that the addition of acetone in the repair materials leads to a significant decrease in the final film thickness with time. This is because acetone has a low viscosity, greatly reducing the initial viscosity of the repair material, expanding its surface tension, and providing a large amount of driving force for the infiltration process.

#### 3.2.3. Freeze–Thaw Cycling Test

**Figure 14:**
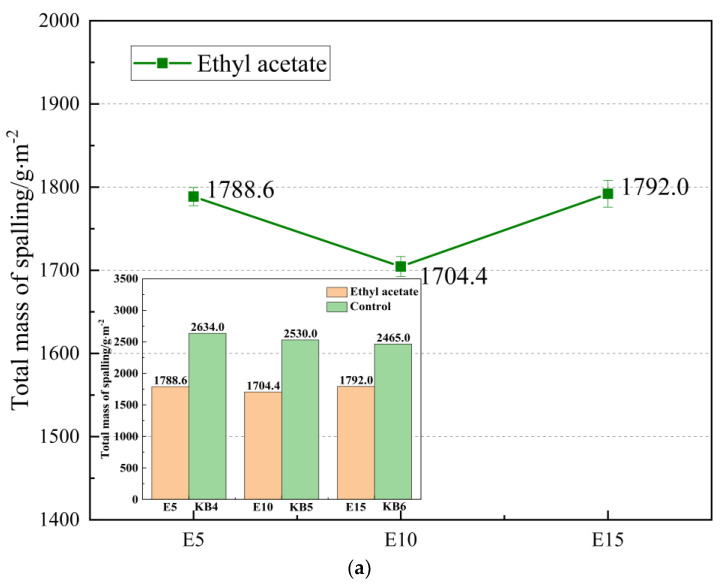

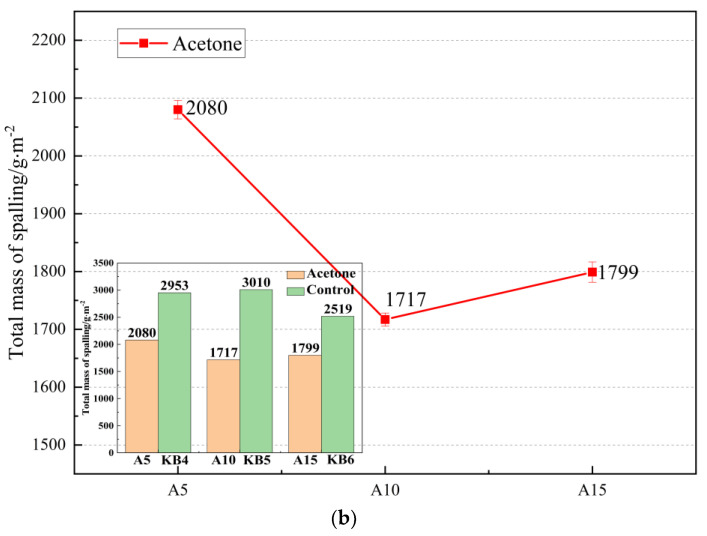
Effect of diluent content on freeze–thaw cycle: (**a**) effect of different contents of ethyl ace- tate on spalling amount; (**b**) effect of different acetone contents on spalling amount.

The influence of the freeze–thaw cycles on the repaired concrete was investigated. By simulating temperature changes, the protective effect of the repair materials on the concrete was tested. The final results were converted into the amount of peeling per square meter as the evaluation standard for the concrete’s resistance to the freeze–thaw cycles. The results are shown in Figure 14a,b.

As shown in Figure 14a,b, it can be seen that the repair material added with 10% ethyl acetate has the best protective performance on concrete, and the average spalling amount per unit area is only 1704.4 g. Adding 5% acetone repair material for concrete protection performance is the worst, with an average spalling amount per unit area of 2080.8 g. Whether it is ethyl acetate or acetone, when the content is 5%, the amount of spalling shows a trend of slow and then fast, and when the content is 15%, the overall spalling speed is faster, which is because the mechanical strength of the repair material itself is significantly reduced, even if the penetration performance is excellent it cannot be well bonded and solidified with concrete, so the amount of solvent added should be between 10–15%.

#### 3.2.4. SEM

**Figure 15:**
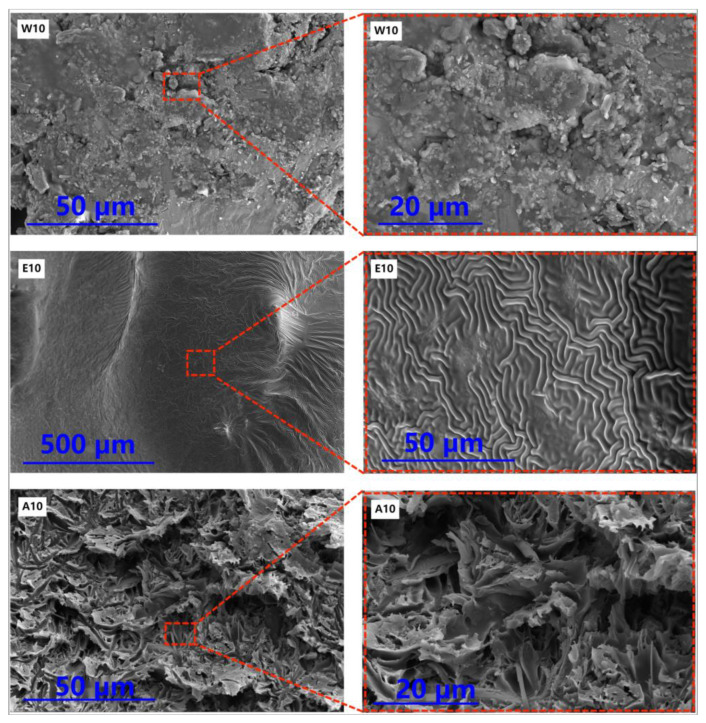
SEM images of three types of diluents.

Samples W10, E10, and A10 were applied to the surface of the concrete and cured for 28 days under natural conditions. Samples were taken from the surface of the broken parts and observed under SEM to investigate the bonding between the repair materials and cement paste. The results are shown in Figure 15.

Figure 15 shows the microstructure of the three samples. In the W10 sample, the repair material mostly remains on the surface of the cement paste, and the mortar surface is very rough and contains a large number of micropores, which provide channels for harmful substances to penetrate the interior of the concrete and do not provide effective protection. For the E10 sample, a polymer film was clearly visible on the surface of the mortar, and the repair material formed a tightly interwoven structure, which is conducive to preventing or delaying the penetration of corrosive media in the mortar. In contrast to the previous two samples, the A10 sample showed pinholes due to the rapid volatilization of solvents [30]. This is because when the repair material is dried, the volatilized solvent will form bubbles on the surface of the material. If the bubbles cannot be eliminated in time, pinhole-like small holes will be left on the surface of the coating.

## 4. Conclusions

1. In general, the optimum amount of curing agent is determined by adjusting the content of the curing agent. Then, by adding diluent, the repair material has good infiltration performance and can be consolidated in a certain depth of concrete surface, forming a “rooted type” filling so that the mechanical strength and durability of the concrete surface can be improved.
2. It was found that the 60-min average viscosity of the repair material increased first and then decreased as the content of DMA added was 0.3%, 0.5%, 0.7%, and 1%. When the reaction time reached 40–60 min, the viscosity of the system increased significantly. The viscosity of the repair material with 1% DMA content reached 284 mPa·s at 60 min, which was 46.12% higher than that of 0.3% DMA on average. Considering the controllability of viscosity and the prevention of explosion, DMA content in the range of 0.3–0.5% is more suitable.
3. After conducting surface-drying time, through-drying time, and bonding strength tests, it was found that the surface-drying time and through-drying time of the repair material with 1% DMA content were 0.8 h and 1.5 h, respectively, which were 60% and 67% shorter than those of the repair material with 0.3% DMA content. However, excessive addition of the curing agent can lead to a sharp increase in the reaction of the repair material, resulting in the formation of tiny bubbles. When the curing agent content was 0.5%, the bonding strength reached its peak, with values of 10.99 MPa and 9.24 MPa at the dry interface and wet interface, respectively. Therefore, the optimal curing agent content was determined to be 0.5%.
4. A comparison study of three different diluents showed that sample E10 had a penetration depth of 24 mm and a film thickness of only 35 µm after 72 h. After the freeze–thaw cycling test, E10 showed the best protective performance for concrete, with an average peeling strength of only 1704.4 g/m². SEM showed that the polymer formed a tightly interwoven protective film on the surface of the cement paste, effectively preventing the ingress of corrosive substances.

## Figures and Tables

**Table 1:** Proportioning of C30 Concrete.

Water	Cement	Sand	Coarse Aggregate
205 kg/$m^{3}$	340 kg/$m^{3}$	705 kg/$m^{3}$	1150 kg/$m^{3}$

**Table 2:** Proportion of raw materials.

NO.	MMA /g	BPO /%	DBP /%	DMA /%	PCE /%	Anhydrous Ethanol /%	Ethyl Acetate /%	Acetone /%
KB	80	1.5	30	0.5	10	0	0	0
W5	80	1.5	30	0.5	10	5	0	0
W10	80	1.5	30	0.5	10	10	0	0
W15	80	1.5	30	0.5	10	15	0	0
E5	80	1.5	30	0.5	10	0	5	0
E10	80	1.5	30	0.5	10	0	10	0
E15	80	1.5	30	0.5	10	0	15	0
A5	80	1.5	30	0.5	10	0	0	5
A10	80	1.5	30	0.5	10	0	0	10
A15	80	1.5	30	0.5	10	0	0	15

## Data Availability

The data presented in this study are available on request from the corresponding author.
